# The obesity paradox in beyond total mesorectal excision surgery for locally advanced and recurrent rectal cancer

**DOI:** 10.1007/s13304-019-00631-6

**Published:** 2019-02-21

**Authors:** Daniel L. H. Baird, Constantinos Simillis, Gianluca Pellino, Christos Kontovounisios, Shahnawaz Rasheed, Paris P. Tekkis

**Affiliations:** 1 Department of Colorectal Surgery, The Royal Marsden Hospital, London, UK; 2 Department of Surgery and Cancer, Imperial College London, Chelsea and Westminster and the Royal Marsden Campus, London, UK; 3 Department of Colorectal Surgery, Chelsea and Westminster Hospital, London, UK

**Keywords:** Rectal cancer, Beyond TME, Body Mass Index

## Abstract

The objective is to investigate preoperative body mass index (BMI) in patients receiving beyond total mesorectal excision (bTME) surgery. The primary end point is length of postoperative stay. Secondary end points are length of intensive care stay, postoperative morbidity and overall survival. BMI is the most commonly used anthropometric measurement of nutrition and studies have shown that overweight and obese patients can have improved surgical outcomes. Patients who underwent a bTME operation for locally advanced or recurrent rectal cancer were put into three BMI (kg/m^2^) groups of normal weight (18.5–24.9), overweight (25–29.9) and obese (≥ 30) for analysis. Included are 220 consecutive patients from a single centre. The overall length of stay, in days ± standard deviation (range), for normal weight, overweight and obese patients was 21.14 ± 16.4 (6–99), 15.24 ± 4.3 (7–32) and 19.10 ± 9.8 (8–62) respectively (*p* = 0.002). The mean ICU length of stay was 5.40 ± 9.1 (1–69), 3.37 ± 2.4 (0–19) and 3.60 ± 2.4 (1–14), respectively (*p* = 0.030). There was no significant difference between the three groups in terms of postoperative morbidity or overall survival. Patients with a normal weight BMI in this cohort have a significantly longer length of stay in ICU and in hospital than overweight or obese patients. This is seen with no significant difference in morbidity or overall survival.

## Introduction

Colorectal cancer is the third most common in the world and 30–40% is in the rectum [1, 2]. 10–20% of rectal cancer patients present with locally advanced disease [3, 4]. Around 10% of curative operations for rectal cancer will be locally recurrent [5] and around half of the patients with recurrent rectal cancer have isolated and potentially curable disease [6]. A beyond total mesorectal excision (bTME) operation offers the best chance of cure for locally advanced and recurrent rectal cancer [7, 8].

The incidence of rectal cancer is increasing [9] and survival is improving [10–12]. Advancing age, male sex and genetic susceptibility are all associated with a worsening in mortality from rectal cancer [13]. There is a known association between lifestyle factors and the incidence of colorectal cancer; however, the mechanisms are poorly understood [13].

Nutrition in surgery is important as malnutrition and obesity can both impact negatively on outcomes. Hiram et al. in 1936 was the first to report on the importance of nutrition in surgery showing a 33% versus 3.5% mortality rate in peptic ulcer surgery in the malnourished and the well nourished, respectively [14]. A nutritional assessment should consist of ‘a comprehensive approach to diagnosing nutrition problems that uses a combination of the following: medical, nutrition, and medication histories; physical examination; anthropometric measurements; and laboratory data’ [15]. Many tools for nutritional assessment have been suggested, with varying definitions of malnutrition; however, there is no agreement on a gold standard [16, 17]. Measuring a patient’s BMI [18] is the most commonly used anthropometric measurement of nutritional status [19]. There is a large variation of BMI thresholds used in the literature [20, 21] and the use of BMI alone as an indicator of nutrition has limitations including not distinguishing between low weight due to fat depletion or muscle depletion [22].

Malnutrition can be created by the systemic effects of cancer or the hosts response to cancer and can be compounded by chemoradiotherapy [23–27]. Patients with gastrointestinal cancers are high risk for presenting with and developing malnutrition as they can create a catabolic effect and cause anorexia, nausea, vomiting, gastrointestinal tract obstruction and malabsorption [28, 29]. 30–60% of colorectal cancer patients and 80% of advanced colorectal cancer patients are reported as malnourished [27, 30]. Malnutrition impacts on morbidity, mortality, length of stay, readmission rates, quality of life and it is considered to negatively affect all bodily functions [16, 17, 25, 27, 31–34].

The World Health Organisation states that worldwide obesity has more than doubled since 1980 [35]. In 2015, National Health Service England reported an increase in the prevalence of obesity noting that 58% of women, 65% of men and 20% of 5 and 6 year olds are overweight or obese [36]. Being overweight or obese is associated with an increase in the incidence of multiple cancers [37, 38] and an increase in overall mortality in the general population [39, 40]. It can make an operation more technically difficult and increase postoperative complications [38, 41–43]. High BMI specifically is an established risk factor for developing colorectal cancer, obese men of all ages are at the greatest risk [44, 45], and the mechanism is unclear [38, 43]. It may be due to a direct biological mechanism such as central obesity, associated with insulin resistance [42, 46], or the creation of a pro-inflammatory state which, amongst other mechanisms, have been implicated in the development of colorectal cancer [41, 43]. It may also be due to indirect mechanisms such as lifestyle choices of being sedentary, eating the wrong foods or smoking [43].

This work aims to examine the impact that preoperative BMI has on postoperative outcomes in locally advanced and recurrent bTME rectal cancer.

## Method

### Inclusion criteria

Consecutive adults undergoing a curative intent bTME operation, as defined by the beyond TME consensus statement [4], for locally advanced primary or recurrent rectal adenocarcinoma, under the care of the senior authors at The Royal Marsden Hospital were included. All patients had a preoperative BMI recorded.

### End points

The primary end point is the effect that BMI has on in-hospital length of stay. Secondary end points are to assess the effect that BMI has on intensive care length of stay, postoperative morbidity and overall survival.

### Definitions

#### Definition of BMI categories

The World Health Organisation (WHO) BMI categories were used in this work; BMI (kg/m^2^): ≤ 18.5 as underweight, 18.5–24.9 as normal weight, 25–29.9 as overweight, and ≥ 30 as obese [18].

#### Definition of operative groups

Pelvic exenteration: a multivisceral resection of pelvic contents to clear central, anterior, posterior, lateral or inferior compartments as is required. bTME other: an operation for a tumour that extends beyond the circumferential resection margin on preoperative imaging.

### Data source

Patients were identified from a database at The Royal Marsden Hospital from January 2006 to December 2016. Computerised records for each patient were retrospectively interrogated. Body Mass Index (BMI) (kg/m^2^) was measured immediately before surgery as part of the preoperative assessment. Clinical outcomes were collected retrospectively from a prospectively kept computerised record of the patient’s admission.

### Treatment

Patient evaluation included a history and examination, endoscopy with a biopsy, a computed tomography scan of the thorax, abdomen and pelvis (CT-TAP) and a pelvic MRI scan. If the tumour had high-risk features or if distant metastasis was suspected, a positron emission tomography (PET) scan was performed. Ongoing management plans were agreed through a specialised bTME multi-disciplinary team (MDT) meeting.

Chemoradiotherapy was given according to European Guidelines; radiation of 45–50.4 Grays in 25–30 fractions over 5 weeks with concomitant chemotherapy of 5-fluorouracil (5-FU)/capecitabine. From 2010, onwards patients were also considered for induction chemotherapy. Decisions regarding adjuvant chemotherapy were made at the MDT.

Surgery was undertaken either immediately or 6–8 weeks after neoadjuvant therapy by a Consultant-led team experienced in complex rectal cancer surgery. Where appropriate, Consultant-led teams in Plastic and Reconstructive surgery, Urology, Gynaecology and Vascular surgery were involved. All patients were admitted to the ICU postoperatively as standard and discharge from the ICU was made by an intensive care consultant.

### Statistics

All statistical comparisons are between the BMI (kg/m^2^) groups of normal weight (18.5–24.9), overweight (25–29.9) and obese (≥ 30). The BMI data were tested for normality using Shapiro–Wilk test. To investigate differences between the BMI groups, the analysis of variance (ANOVA) test was used. For survival analysis, the Kaplan–Meier method was used and comparison between the groups was with the log rank Mantel–Cox test for significance. Estimated mean survival is given along with the 95% confidence intervals (95% CI). All statistical analysis was considered significant with a *p* value of 0.05 or less.

## Results

320 patients had undergone a bTME resection, as defined by the bTME consensus statement [4], 52 of which included a sacrectomy under the senior authors. 4 palliative resections, 35 non-adenocarcinoma tumours and 61 patients from outside of our institution were excluded.

Included are 220 consecutive patients. 179 (81.4%) patients received neoadjuvant chemoradiotherapy, 6 (2.7%) received chemotherapy only, 4 (1.8%) received radiotherapy only and 31 (14.1%) had no neoadjuvant therapy. 106 (48.2%) patients had adjuvant therapy. 151 patients underwent a pelvic exenteration and 34 of them had an en bloc sacrectomy. 69 patients had a ‘bTME other’ operation that included 14 recurrent rectal cancers, 14 synchronous resections, 10 with MRI predicted pelvic sidewall involvement, 26 and 5 with involved and threatened circumferential resection margins, respectively.

Follow-up time ranged from 1.5 to 119.6 months with a median follow-up time of 26.0 months. The 3-year disease-free survival is 66% and the 5-year overall survival is 71%.

The BMI data were tested using Shapiro–Wilk test which showed the data to be normally distributed (*p* < 0.0001). Each BMI category of normal weight, overweight and obese was also tested separately and they are all normally distributed (*p* = 0.005, *p* < 0.0001 and *p* < 0.0001, respectively).

The BMI (kg/m^2^) categories of normal weight (18.5–24.9), overweight (25–29.9) and obese (≥ 30) had 81, 97 and 42 patients, respectively. The mean BMI for the whole cohort ± standard deviation (± SD) (range) was 26.3 ± 4.3 (18.5–43) kg/m^2^.

Patient demographics are shown in Table 1. There are 138 (62.7%) males and 82 (37.3%) females. When broken into the normal weight, overweight and obese categories, significantly, more males were overweight and obese (*p* = 0.004). There was no significant difference between the BMI groups in terms of age (*p* = 0.933) or ASA grade (*p* = 0.263).

**Table 1:** Demographics

Factor	bTME results	BMI 18.5–24.9	BMI 25–29.9	BMI ≥ 30	ANOVA
Category	Total 220 cases	Total 81 cases	Total 97 cases	Total 42 cases	*p* value
Gender, *n* (%)					
Male	138 (62.7)	39 (48.1)	68 (70.1)	31 (73.8)	0.004
Female	82 (37.3)	42 (51.9)	29 (29.9)	11 (26.2)	
Age in years, mean ± SD (range)	61.70 ± 12.5 (28–89)	61.48 ± 13.5 (28–89)	62.04 ± 12.7 (27–85)	61.98 ± 10.25 (40–80)	0.933
ASA					
I + II	189 (85.9)	67 (82.7)	84 (86.6)	38 (90.5)	0.263
III + IV	31 (14.1)	14 (17.3)	13 (13.4)	4 (9.5)	

*ASA* American Society of Anaesthesiologists, *BMI* body mass index (kg/m^2^), *bTME* beyond total mesorectal excision, *ANOVA* analysis of variance, *SD* standard deviation, *n* number of patients

There was no significant difference between the BMI groups in terms of neoadjuvant treatment received (see Table 2). In the group of 31 patients who went straight to surgery, 17 were normal weight, 11 were overweight and 3 were obese which was significantly (*p* = 0.048) different. There was no significant difference between the groups in terms of extent of surgery (*p* = 0.767), 30-day morbidity (*p* = 0.461), overall morbidity (*p* = 0.563) or morbidity in terms of Clavien–Dindo classification (see Table 2). There was no 90-day mortality in any patients.

**Table 2:** Treatment, postoperative morbidity, length of stay, preoperative tumour and postoperative pathology

Factor	bTME results	BMI 18.5–24.9	BMI 25–29.9	BMI ≥ 30	ANOVA
Category	Total 220 cases	Total 81 cases	Total 97 cases	Total 42 cases	*p* value
Oncological treatment, *n* (%)					
Neoadjuvant chemoradiation	179 (81.4)	61 (75.3)	81 (83.5)	37 (88.1)	0.158
Neoadjuvant chemotherapy	6 (2.7)	1 (1.2)	3 (3.1)	2 (4.8)	0.473
Neoadjuvant radiotherapy	4 (1.8)	2 (2.5)	2 (2.1)	0 (0.0)	0.619
No chemoradiotherapy	31 (14.1)	17 (21.0)	11 (11.3)	3 (7.1)	0.048
Adjuvant therapy	106 (48.2)	35 (43.2)	51 (52.6)	20 (47.6)	0.378
Type of operation, *n* (%)					
Exenterative operation	151 (68.6)	53 (65.4)	69 (71.1)	29 (69.0)	0.767
bTME other	69 (31.4)	28 (34.6)	28 (28.9)	13 (31.0)	
Postoperative morbidity					
30 days, *n* (%)	87 (39.5)	34 (42.0)	34 (35.1)	19 (45.2)	0.461
All during admission, *n* (%)	110 (50.0)	38 (46.9)	48 (49.5)	24 (57.1)	0.563
Clavien–Dindo classification					
I	11 (5.0)	2 (2.5)	6 (6.2)	3 (7.1)	0.358
II	58 (26.4)	17 (21.0)	29 (29.9)	12 (28.6)	0.380
IIIa	10 (4.5)	3 (3.7)	4 (4.1)	3 (7.1)	0.601
IIIb	17 (7.7)	7 (8.6)	6 (6.2)	4 (9.5)	0.807
IVa	10 (4.5)	6 (7.4)	2 (2.1)	2 (4.8)	0.294
IVb	4 (1.8)	3 (3.7)	1 (1.0)	0 (0.0)	0.295
V	0 (0.0)	0 (0.0)	0 (0.0)	0 (0.0)	
Length of stay in days, mean ± SD (range)					
ICU	4.1 ± 6.5 (0–69)	5.40 ± 9.1 (1–69)	3.37 ± 2.4 (0–19)	3.60 ± 2.4 (1–14)	0.030
In hospital	18.1 ± 11.5 (6–99)	21.14 ± 16.4 (6–99)	15.24 ± 4.3 (7–32)	19.10 ± 9.8 (8–62)	0.002
Locally advanced primary cancer	171 (77.7)	68 (84.0)	73 (75.3)	30 (71.4)	0.087
Recurrent rectal cancer	49 (22.3)	13 (16.0)	24 (24.7)	12 (28.6)	
Pathological T staging, *n* (%)					
pT0–pT2	52 (23.6)	19 (23.5)	26 (26.8)	7 (16.7)	0.341
pT3	90 (40.9)	33 (40.7)	40 (41.2)	17 (40.5)	0.808
pT4	57 (25.9)	22 (27.2)	19 (19.6)	16 (38.1)	0.096
pT unknown	21 (9.6)	7 (8.6)	12 (12.4)	2 (4.7)	0.29
Pathological N staging, *n* (%)					
pN0	136 (61.8)	53 (65.4)	56 (57.7)	27 (64.3)	0.448
pN1	51 (23.2)	16 (19.8)	24 (24.7)	11 (26.2)	0.666
pN2	15 (6.8)	6 (7.4)	6 (6.2)	3 (7.1)	0.983
pN unknown	18 (8.2)	6 (7.4)	11 (11.3)	1 (2.4)	0.161
Complete resection, *n* (%)	198 (90.0)	74 (91.4)	86 (88.7)	38 (90.5)	0.725
Lymph node, mean ± SD (range)					
Total yield	16.3 ± 11.4 (1–62)	18.0 ± 13.2 (1–62)	15.5 ± 10.4 (1–56)	14.7 ± 10.0 (1–52)	0.288
Number of positive nodes	1.2 ± 3.4 (0–26)	1.32 ± 4.2 (0–25)	0.89 ± 1.8 (0–10)	1.51 ± 4.5 (0–26)	0.626

*ICU* intensive care unit, *bTME* beyond total mesorectal excision, *ANOVA* analysis of variance, *BMI* body mass index (kg/m^2^), *SD* standard deviation, *n* number of patients

The mean ICU length of stay in days, ± SD (range) was 5.4 ± 9.1 (1–69), 3.37 ± 2.4 (0–19) and 3.6 ± 2.4 (1–14) for normal weight, overweight and obese BMI categories, respectively which was statistically significant (*p* = 0.030). The overall length of stay was 21.14 ± 16.4 (6–99), 15.24 ± 4.3 (7–32) and 19.1 ± 9.8 (8–62) for normal weight, over weight and obese BMI categories, respectively, which was statistically significant (*p* = 0.002) (see Table 2).

There was no statistically significant difference between the three BMI groups in patients who presented with a locally advanced primary versus recurrent rectal cancer, the postoperative histopathological T and N staging, the completeness of the resection, the overall number of lymph nodes and the number of cancer positive lymph nodes in the specimen (see Table 2).

**Fig. 1:**
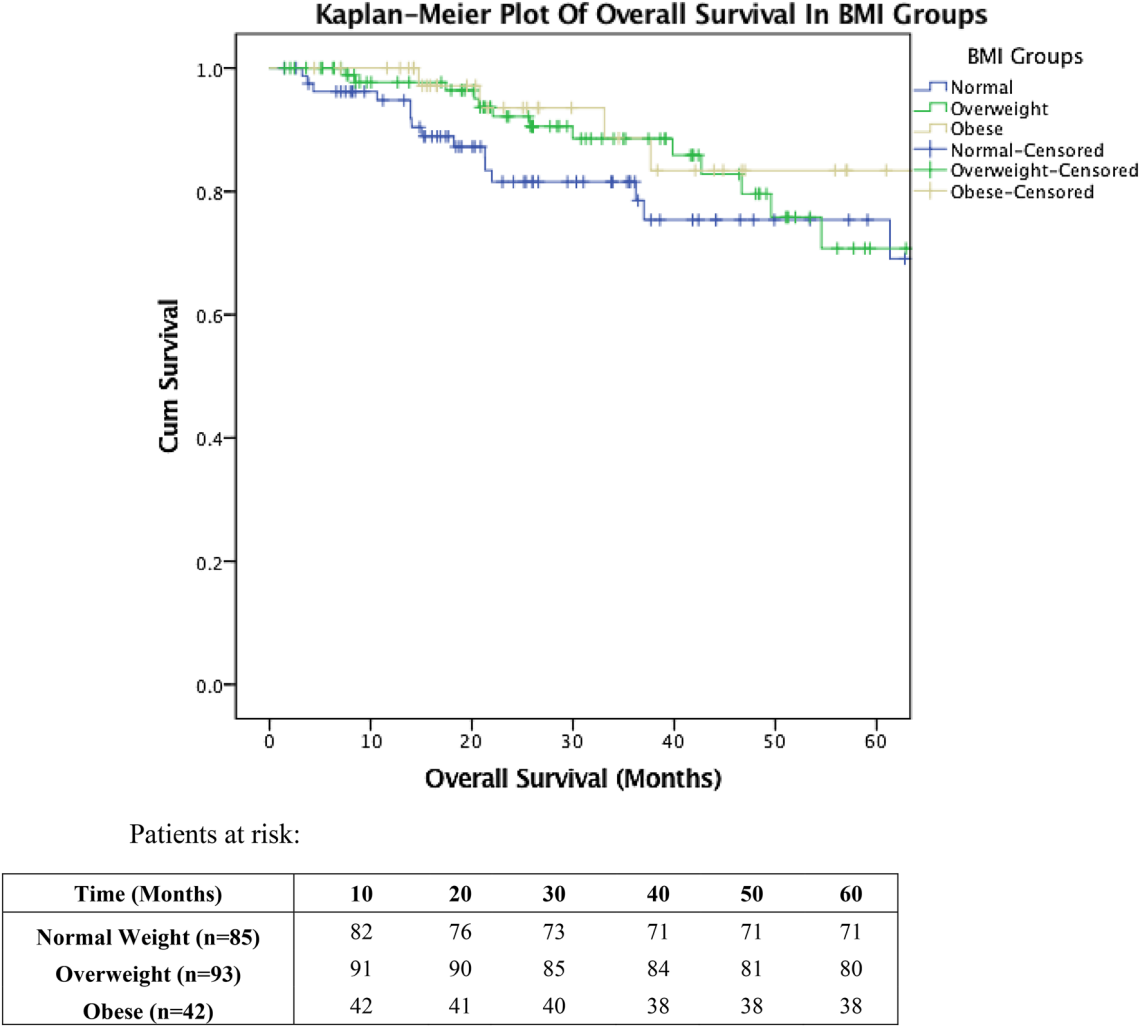
Kaplan–Meier plot showing overall survival vs. BMI by category. Log rank Mantel–Cox test for significance: *p* = 00.283

Mean overall survival was 67.73 (95% CI 59.88–75.57) months, 95.44 (95% CI 82.88–108.01) months and 68.63 (95% CI 61.43–75.83) months for normal weight, over weight and obese BMI categories, respectively, which was not statistically significant (*p* = 0.283) (see Table 3; Fig. 1).

**Table 3:** Overall survival against BMI in normal, overweight and obese categories

BMI	Number of patients, *n* (%)	Death, *n* (%)	Mean survival (months)	95% Confidence interval	*p* value
18.5–24.9	85 (38.6)	16 (7.3)	67.73	59.88–75.57	0.283
25–29.9	93 (42.3)	13 (5.9)	95.44	82.88–108.01	
≥ 30	42 (19.1)	4 (1.8)	68.63	61.43–75.83	

*BMI* body mass index (kg/m^2^), *n* number of patients

*p* value calculated by Kapla–Meier method and log rank test

## Discussion

It is surprising how heterogeneous the literature is on the issue of BMI and surgical outcomes in general which is further complicated by a large variation used in BMI cut offs and definitions [34, 47–51]. There is little written about the relationship between BMI and postoperative outcomes in bTME surgery for locally advanced and recurrent rectal cancer. Mullen et al. [48] conducted a multicentre study with 118,707 non-bariatric, general surgery patients investigating the relationship between BMI and morbidity and mortality. Mullen et al. [48] noted a reverse J-shaped relationship where being underweight (BMI < 18.5 kg/m^2^) suffered the most morbidity and mortality followed by being morbidly obese (BMI > 40 kg/m^2^). The least morbidity and mortality were seen in the overweight (18.5–25 kg/m^2^) and obese (25–30 kg/m^2^) groups [48]. Other studies have also seen an apparent protective effect of being overweight or obese, as opposed to being underweight or morbidly obese, which has been labelled as ‘the obesity paradox’ [48, 50–52]. The obesity paradox has been demonstrated in a variety of areas including colorectal cancer [34, 52, 53], critically ill patients [54], renal failure [55], heart failure [56] coronary artery disease patients undergoing intervention [51] and general surgery patients [48]; however, the reason for the observed effect is not clear.

Burden et al. [32] reported on 87 UK patients with colorectal cancer, who underwent surgery, and the preoperative mean BMI ± SD was 26.6 ± 5.1 kg/m^2^. Read et al. [57] reported on 51 patients with advanced colorectal cancer who had a median BMI (range) of 27 (17–41) kg/m^2^. Beaton et al. [52] looked at 31 patients with colorectal cancer undergoing pelvic exenteration with a mean BMI ± SD of 24.3 ± 5.9 kg/m^2^. The mean BMI in this cohort, ± SD (range) was 26.3 ± 4.3 (18.5–43) kg/m^2^. McWhirter et al. [22] reported that in a UK hospital, 23% of men and 28% of women admitted for any reason have a BMI of less than 20 kg/m^2^. In this cohort, 15 (6.8%) patients had a BMI of less than 20 kg/m^2^. It is interesting to note that this cohort did not see large amounts of low BMI patients, despite all being either locally advanced or recurrent rectal cancer; these data do, however, match up with previously published data on BMI in colorectal cancer [22, 32, 52, 57].

There are significantly more overweight and obese males in this cohort, which would be expected as obesity rates are higher in males across the general United Kingdom population [36]. Furthermore, being male and overweight or obese are risk factors for developing colorectal cancer [37, 38]. This male predominance may be related to central adiposity which is more common in males and is linked with metabolic abnormalities which is thought to be a risk factor for developing colorectal cancer [46].

### Morbidity

Morbidity rates after resection for locally advanced and recurrent rectal cancer are expected to be high due to the extent and complexity of the surgery. In bTME surgery, all-cause morbidity is expected to be above 50% [58] and a range between 35–78% is quoted in the literature [53, 58–61]. It is also expected that morbidity rates after surgery for recurrent rectal cancer would be worse [61]. Moghadamyeghaneh et al. [58] reported that after pelvic exenteration, 65.7% experienced morbidity, with infective (42.6%) and haemorrhagic (39.0%) complications being the two largest causes. Healy et al. [53] reported on 414 patients with colorectal cancer and found no significant difference in the overall postoperative morbidity between BMI groups. Healy et al. [53] reported that patients with a BMI arbitrary cut-off point of less than 20 kg/m^2^ had significantly more ‘major complications’ defined as pneumonia, acute respiratory distress syndrome, abdominal or pelvic abscesses, organ failure and myocardial infarction [53]. In this cohort, 87 (39.5%) and 110 (50.0%) patients experienced morbidity in the first 30 days or during the whole admission, respectively. This work did not associate being normal weight, overweight or obese was with any significant difference in postoperative morbidity in the first 30 days, during the whole admission or after being categorised into the Clavien–Dindo classification.

### Survival

Overall survival in this cohort was not significantly different across the groups of normal weight, overweight and obese, but it is interesting to note that normal weight had the worst overall survival (see Fig. 1). Healy et al. [53] also noted a non-significantly worse survival in the non-obese group across a colorectal cancer cohort [53]. Mullen et al. [48] reported that overweight and obese general surgery patients have a significantly lower crude and adjusted mortality rate compared to normal weight patients [48]. This finding is unexpected and is contrary to the general population where studies have shown that an increased BMI or having an overweight or obese status is linked with increased mortality [39, 40]. The BMI measurements in this study are a single snap shot and will not have accounted for recent weight loss. It has been shown previously that colorectal cancer patients, with a normal BMI, can be malnourished [32, 57]. It is conceivable that patients in the normal BMI range may have recently lost weight and may be in a state of malnourishment.

### Length of stay

Mullen et al. [48] reported a significant difference in the mean length of stay between underweight (BMI < 18.5 kg/m^2^), and obese (BMI 30–35 kg/m^2^) patients of 9.1 days and 4.1 days, respectively [48]. Beaton et al. [52] reporting on exenterative patients, also noted a significant difference in the mean length of stay of 50 days if underweight (< 18.5 kg/m^2^), 16.0 days if normal weight (18.5–25 kg/m^2^) and 14 days if overweight/obese (> 25 kg/m^2^) [52]. Garth et al. [31] undertook a comprehensive assessment of nutrition in patients with upper gastrointestinal and colorectal cancer and reported a doubling of the length of stay in malnourished patients [31]. These data show a significantly longer length of stay in ICU and overall for patients in the normal weight as compared to overweight or obese categories, without significantly more morbidity which has been shown before [31, 48, 52, 57]. This work is not able to define the causality of this finding; however, a state of malnutrition may be implicated.

### Limitations

This is a retrospective single-centre analysis. Patients undergoing bTME surgery are carefully considered which introduces a selection bias. This work looks at the impact that a single variable, measured on a single occasion, has on outcomes that are multifactorial. BMI as a single measure of nutrition is likely to be inadequate in comparison to other multifaceted measurement tools. Further data points on preoperative comorbidities beyond ASA status or intraoperative data points on the complexity or length of surgery and reconstruction would have been useful to examine these endpoints further and should be included in any further work.

## Conclusion

The BMI, especially in isolation, as a measure of nutritional status has drawbacks but is commonly used. This work found that normal weight patients have a significantly longer length of ICU and overall hospital stay compared to overweight and obese patients. This was seen despite no significant difference in postoperative morbidity or overall survival. This study adds evidence to the existence of an obesity paradox in bTME rectal cancer but cannot illuminate upon causality.

The message from this work and other studies is contrary to the thinking that overweight or obese colorectal patients are a high surgical risk; in fact, the opposite appears to be true. Further work is required to better define and investigate this area.
